# The Therapeutic Value of Kidney Extract

**Published:** 1904-06-18

**Authors:** 


### THE THERAPEUTIC VALUE OF KIDNEY EXTRACT.

M. J. Renaut[1] reports four cases of kidney disease with uræmic symptoms successfully treated by the administration of a watery extract of fresh pig's kidneys. One patient suffered from albuminuria and cardiac degeneration after influenza, two from interstitial nephritis, and the fourth from mixed nephritis. The extract is prepared by macerating the cortex of the kidneys in water for four hours and decanting the clear liquid, and the equivalent of two to three kidneys should be taken daily. After 10 days' treatment the administration should be suspended for several days, otherwise skin eruptions and other disturbances may ensue. When recovery takes place it is wise to give some of the extract at occasional intervals. In the cases recorded the patients were in good health 12 months after the treatment was commenced.

[1] Bull. Gén. de Thérap., 1904.

